# Association of serum chromium levels with malnutrition in hemodialysis patients

**DOI:** 10.1186/s12882-019-1476-x

**Published:** 2019-08-05

**Authors:** Ching-Wei Hsu, Cheng-Hao Weng, Cheng-Chia Lee, Tzung-Hai Yen, Wen-Hung Huang

**Affiliations:** 1 Department of Nephrology and Clinical Poison Center, Chang Gung Memorial Hospital, Linkou Medical Center, Taoyuan, Taiwan; 2 Chang Gung University College of Medicine, Taoyuan, Taiwan; 3 Department of Nephrology and Clinical Poison Center, Chang Gung Memorial Hospital, 199, Tung-Hwa North Road, Taipei, Taiwan, Republic of China

**Keywords:** Chromium, End stage renal disease, Hemodialysis, Nutrition

## Abstract

**Background:**

Chromium is an essential trace metal that reduces oxidative stress and inflammation. In patients undergoing maintenance hemodialysis (MHD), a correlation among chromium exposure, inflammation, and malnutrition remains unclear. This study examined the possible effects of serum chromium levels (SCLs) in MHD patients.

**Methods:**

Initially, 732 MHD patients in dialysis centers were recruited. A total of 647 patients met the inclusion criteria and were stratified by SCL into four equal-sized groups: first quartile (< 0.29 μg/L), second quartile (0.29–0.56 μg/L), third quartile (0.57–1.06 μg/L), and fourth quartile (> 1.06 μg/L). Demographic, biochemical, and dialysis-related data were obtained for analyses. The analysis included nutritional and inflammatory markers.

**Results:**

As compared with the highest quartile group, more subjects in the lowest quartile group were of an older age; had lower hemoglobin and creatinine levels; had a higher prevalence of DM and malnutrition (serum albumin level < 3.6 g/dL); and higher serum transferrin saturation and ferritin levels. A stepwise multiple linear regression analysis revealed a significant negative correlation between malnutrition and SCL (β coefficient = − 0.129, *p* = 0.012) and negative associations among body mass index (β coefficient = − 0.010, *p* = 0.041), ferritin (β coefficient = − 0.107, *p* = 0.001) and SCL. A multivariate logistic regression analysis also demonstrated a negative correlation between malnutrition and SCL. With a 10-fold increase in SCL, the risk ratio of malnutrition was 0.49 (95% confidence interval: 0.25–0.96; *p* = 0.039).

**Conclusions:**

SCL is significantly associated with malnutrition in MHD patients. Further evaluation of the relationship between clinical outcomes (morbidity/mortality) and SCL is necessitated.

## Background

Studies have suggested that trivalent chromium is an essential nutrient; potentiates insulin action in peripheral tissue; and is essential for lipid, protein, and fat metabolism in animals and humans [1, 2]. The lower plasma chromium level is associated with hyperglycemia, insulin resistance, high inflammatory status and increased cardiovascular risk in humans [2]. The compounds containing *hexavalent* chromium are widely known to have mutagenic and carcinogenic effects when inhaled or orally ingested in large amount [3, 4]. However, not all studies have reported beneficial effects of nutritional supplement with chromium [5, 6].

Chromium has been shown to accumulate in the bones of patients with end-stage renal disease (ESRD), and increased serum chromium levels (SCLs) have been found in patients undergoing maintenance hemodialysis (MHD) [7]. However, no study mentioned the clinical advantages or disadvantages of an elevated SCL in patients undergoing MHD until now.

Although traditional risk factors for cardiovascular disease are common in ESRD patients, recent clinical studies indicated that chronic inflammation and malnutrition may cause protein-energy wasting in ESRD patients and poor short-term survival in this population [8, 9]. Correcting inflammation, malnutrition, and even protein-energy wasting may reduce mortality for patients undergoing MHD. Therefore, identifying correctable factors associated with malnutrition, inflammation, or both is important for these patients. We therefore conducted a cross-sectional study to evaluate the relationship among SCLs, malnutrition, and inflammation using details from clinical examinations of MHD patients.

## Methods

### Patients

Patients were identified using the following inclusion criteria: a minimum age of 18 years and on MHD more than 6 months. Initially, we enrolled 732 patients from the dialysis units of our hospital. Patients with the following conditions were excluded: malignancies (*n* = 10), obvious infectious diseases (*n* = 12), hospitalization or undergone surgery within 3 months before the study (*n* = 32). Patients were also excluded if they had ever exposed to relatively high chromium levels, including occupational exposure (*n* = 28) and living in heavily contaminated areas (*n* = 3). Finally, this study included a total of 647 MHD patients. The majority of patients underwent 3 HD sessions of 4 h per week. The patients used synthetic polysulfone dialysis membrane and bicarbonate dialysate. No dialyzer reuse is practiced. The following data were collected from dialysis charts and/or electronic medical records: demographics (age and gender), relevant comorbidities (cardiovascular diseases [CVDs], hypertension and diabetes mellitus [DM]), dialysis-related data and biochemical data.

### Measurement of serum chromium levels and biochemical parameters

To ensure that the patients’ samples were not contaminated with chromium during HD, we collected the samples of water and dialysate by using chromium-free plastic bottles. There are 6 dialysis units in our hospital and we collected the samples of water and dialysate in each unit randomly. Therefore, 12 samples of water and dialysate were analyzed for chromium concentration. All of the blood samples of patients were collected just before the start of the mid-week HD session. The samples were drawn in syringes and needles which were batch-tested and shown not to be contaminated with heavy metals. We measured the SCLs following a previously described method [10]. Briefly, 900 μL of modifier solution (HNO_3_ plus Triton X-100) in deionized water and 100 μL of serum or 100 μL of HNO_3_ and 900 μL of dialysate were added to a 1.5-mL Eppendorf tube and mixed. After overnight refrigeration, the vessels were warmed to room temperature and whirl-mixed for 5–10 s, and then centrifuged for 5 min at 11,500 rpm. The supernatants of diluted serums and dialysate samples were transferred to graphite furnace sampler cups. The chromium concentrations were determined by *atomic absorption spectrophotometer* (SpectrAA-220Z; Varian, USA), with the detection limit of 0.05 μg/L. We detected the serum levels of high-sensitivity C-reactive protein (hsCRP) by immunonephelometry (Nanopia CRP; Daiichi Inc., Tokyo, Japan), with a detection limit of 0.15 mg/L. We used the *automated biochemical analyzers* to determine the concentrations of all other biochemical parameters. We calculated the protein catabolism rate (nPCR) using validated equations and the data were normalized to the actual body weight [11]. We also evaluated the clearance of urea in study patients and the data were expressed as Kt/V [12].

For statistical analysis, all participations were divided into 4 equal-sized groups according to the SCL quartile: first (< 0.29 μg/L, *n* = 164), second (0.29–0.56 μg/L, *n* = 159), third (0.57–1.06 μg/L, *n* = 163), and fourth (> 1.06 μg/L, *n* = 161) SCL quartiles.

### Definition of malnutrition and inflammation

To determine the associations between SCLs and the inflammatory or nutritional status, we evaluated the serum albumin and hsCRP levels in different subgroups. MHD patients were considered to have inflammation if they exhibited an hsCRP level of > 3 mg/L, which was correlated with elevated cardiovascular risk in the general population [13, 14]. Individuals with an albumin level of < 3.6 g/dL were considered to be malnourished. This albumin level was close to the lower limit (i.e., 3.5 g/dL) in our hospital and corresponded to the tenth percentile from the Third National Health and Nutrition Examination Survey of Americans [9, 15].

### Statistical analysis

We applied a Kolmogorov–Smirnov test to determine the distribution of variables. *Continuous variables* with normal distribution were *presented as mean* ± standard deviation and non-normal *variables* were *shown* as median with interquartile range (IQR). Categorical variables are presented as number with percentage. We used a trend test to compare the 4 study groups and a *p* value of < 0.05 was considered significant. Non-normally distributed continuous variables such as SCL, intact parathyroid hormone (iPTH), ferritin, and hsCRP were logarithmically converted. We used Pearson correlation coefficient to assess the association among clinical variables. To determine the correlations between log SCL and baseline variables, we applied univariate and multivariate linear regression analyses to obtain the predictive power, which were presented as β coefficient with *standard error (SE).* All potential variables (*p* < 0.05) from the simple linear regression analysis were entered into forward stepwise multiple linear regression analysis. To evaluate variables related to malnutrition and inflammation, we performed univariate and multivariate logistic regression analyses to obtain the odds ratio (OR) and 95% confidence interval (CI). All potential variables (*p* < 0.05) found to be significant in the univariate logistic regression analysis were included in the forward stepwise multivariate logistic regression analysis. Data analyses were performed using Statistical Package for Social Sciences (SPSS), Version 18.0 for Windows (SPSS Inc., Chicago, IL, USA).

## Results

### Study population characteristics

A total of 647 MHD patients (331 men and 316 women) with a mean HD vintage (length of time on dialysis) of 9.5 ± 6.1 years met the inclusion criteria and were enrolled in this study. Table 1 lists their clinical characteristics including age, gender, and body mass index, along with the biological, hematological, and biochemical data. The mean patient age was 57.9 ± 12.2 years, and their biochemical data were as follows: median SCL, 0.57 μg/L (IQR: 0.29, 1.06); mean serum albumin level, 3.98 ± 0.33 g/dL; and median hsCRP level, 2.59 mg/L (IQR: 1.21, 7.04). Table 1 also lists the patient characteristics for the 4 subgroups. Compared with the second SCL quartile (*n* = 159; median SCL, 0.39 μg/L; IQR: 0.34, 0.46), third SCL quartile (*n* = 163; median SCL, 0.80 μg/L; IQR: 0.65, 0.92), and fourth SCL quartile (*n* = 161; median SCL, 1.59 μg/L; IQR: 1.26, 2.26) groups, the first SCL quartile group (*n* = 164; median SCL, 0.20 μg/L; IQR: 0.14, 0.24) seemed to comprise patients with an older age, lower hemoglobin and creatinine levels and higher prevalence of DM and malnutrition, as well as higher serum transferrin saturation and ferritin levels (Table 1). The groups did not differ in terms of gender, smoking status, body mass index, use of a fistula as a blood access, biocompatible membrane dialyzers, hypertension history, CVD, HD vintage, Kt/V (Daugirdas), and nPCR. Moreover, the groups did not differ in terms of serum albumin; cholesterol; triglyceride; corrected calcium and phosphate levels; iPTH; hsCRP, the presence of viral hepatitis B antigen and viral hepatitis C antibody.

**Table 1:** Baseline characteristics of studied patients by quartile of SCLs (*n* = 647)

Characteristics	Total patients (*n* = 647)	1st quartile (< 0.29 μg/L) (*n* = 164)	2nd quartile (0.29–0.56 μg/L) (*n* = 159)	3rd quartile (0.57–1.06 μg/L) (*n* = 163)	4th quartile (> 1.06 μg/L) (*n* = 161)	*p*
*Demographics*						
Age (years)	57.9 ± 12.2	59.9 ± 12.1	58.5 ± 11.7	57.3 ± 11.2	55.9 ± 12.7	0.002
Female sex	316 (48.8)	81 (49.3)	88 (55.3)	78 (47.9)	69 (42.9)	0.125
Body mass index (kg/m^2^)	22.1 ± 3.4	22.4 ± 3.7	22.4 ± 3.2	22.2 ± 3.7	21.7 ± 3.2	0.062
Smoking (Yes)	76 (11.7)	10 (6.1)	4 (2.5)	9 (5.5)	13 (8.1)	0.320
*Co-Morbidity*						
Diabetes mellitus (Yes)	195 (30.1)	58 (35.4)	51 (32.1)	45 (27.6)	41 (25.5)	0.036
Hypertension (Yes)	62 (9.6)	16 (9.8)	18 (11.3)	13 (8.0)	15 (9.3)	0.654
Previous CVD (Yes)	50 (7.7)	16 (9.8)	8 (5.0)	10 (6.1)	16 (9.9)	0.780
*Dialysis Related Data*						
Hemodialysis vintage (years)	9.5 ± 6.1	9.2 ± 5.8	9.2 ± 5.8	9.4 ± 6.3	10.3 ± 6.5	0.119
Erythropoietin (U/kg/week)	61.14 ± 47.1	65.5 ± 50.8	60.12 ± 45.9	60.9 ± 46.1	58.0 ± 45.4	0.531
Fistula as blood access (Yes)	496 (76)	120 (73.2)	122 (76.7)	122 (74.8)	132 (82.0)	0.093
Biocompatible membrane dialyzers (Yes)	556 (85.9)	145 (88.4)	141 (88.1)	122 (74.8)	148 (91.9)	0.398
Kt/V (Daugirdes)	1.7 ± 0.3	1.74 ± 0.30	1.81 ± 0.32	1.78 ± 0.31	1.78 ± 0.33	0.510
nPCR (g/kg/day)	1.21 ± 0.37	1.21 ± 0.37	1.21 ± 0.37	1.23 ± 0.37	1.20 ± 0.41	0.838
Residual daily urine of > 100 mL	202 (31.2)	52 (32.5)	50 (31.4)	56 (36.6)	44 (27.3)	0.538
*Biochemical Data*						
Hemoglobin (g/dL)	10.32 ± 1.22	10.14 ± 1.27	10.24 ± 1.21	10.38 ± 1.19	10.53 ± 1.20	0.003
Albumin (g/dL)	3.98 ± 0.33	3.94 ± 0.32	3.98 ± 0.34	4.01 ± 0.34	3.99 ± 0.32	0.087
Albumin of < 3.6 g/dL	75 (11.5)	26 (20.6)	19 (11.9)	17 (10.4)	13 (8.1)	0.019
Creatinine (mg/dL)	10.99 ± 2.38	10.8 ± 2.6	10.8 ± 2.2	11.1 ± 2.4	11.3 ± 2.4	0.020
Transferrin saturation (%)	26.3 ± 11.4	28.0 ± 12.6	26.8 ± 10.9	26.9 ± 12.0	23.8 ± 10.0	0.003
Ferritin (μg/L)	260.7 (79.8, 467.3)	321.7 (110.8, 504.6)	336.6 (99.2, 480.5)	257.0 (71.2, 487.1)	144.4 (53.6, 383.5)	0.001
Corrected-calcium (mg/dL)	9.86 ± 0.93	9.7 ± 0.9	10.0 ± 1.0	9.9 ± 0.8	9.8 ± 1.0	0.236
Phosphate (mg/dL)	4.82 ± 1.31	4.6 ± 1.3	4.9 ± 1.2	4.8 ± 1.2	4.9 ± 1.4	0.137
Intact parathyroid hormone (pg/mL)	239.1 (88.8, 444.4)	216.2 (80.4 408.6)	249.1 (86.9, 423.5)	230.3 (98.6, 472.7)	248.1 (103.0, 475.3)	0.229
HsCRP (mg/L)	2.59 (1.21, 7.04)	2.88 (1.28, 8.93)	2.51 (1.07, 6.16)	2.71 (1.36, 6.34)	2.40 (1.25, 7.24)	0.296
HsCRP of > 3.0 mg/dL	296 (45.7)	78 (47.6)	69 (43.3)	78 (47.9)	71 (44.1)	0.732
*Cardiovascular Risks*						
Cholesterol (mg/dL)	171.7 ± 37.3	170.3 ± 35.1	174.1 ± 39.3	172.8 ± 38.2	170.0 ± 36.7	0.865
Triglyceride (mg/dL)	142.4 ± 97.3	141.6 ± 96.8	142.0 ± 103.7	147.6 ± 105.6	138.6 ± 82.1	0.924
Serum chromium (μg/L)	0.57 (0.29, 1.06)	0.20 (0.14, 0.24)	0.39 (0.34, 0.46)	0.80 (0.65,0.92)	1.59 (1.26, 2.26)	< 0.001

Notes: Data presented as mean ± standard deviation, number (percentage), and median (interquartile range). A *p* of < 0.05 represented significant trends among the groups. Hypertension was defined as blood pressure ≥ 140/90 mmHg based on at least two measurements or regular use of antihypertensive drugs. Diabetes mellitus was diagnosed by a physician previously or by 2 measurements of fasting glucose of 126 mg/dL or more. CVD included cerebrovascular disease, coronary arterial disease, congestive heart failure, and peripheral vascular disease

Abbreviations: *CVD* cardiovascular disease, *hsCRP* high sensitivity C-reactive protein, *nPCR* normalized protein catabolic rate, *SCL* serum chromium level

### Water and dialysate chromium levels

The chromium levels of all the water and dialysate samples (*n* = 12) were < 0.1 μg/L and were far below the American Association for Advancement of Medical Instrumentation (AAMI) standards (chromium, < 14 μg/L).

### Determinants of SCLs in MHD patients

In simple linear regression analysis, we found that log SCL were positively associated with HD vintage, hemoglobin and serum creatinine levels, but negatively associated with age, body mass index, DM, malnutrition status, log ferritin level, and transferrin saturation (*p* < 0.05). After adjusting for potential variables, we revealed that log SCL was *significantly* and negatively associated body mass index (β coefficient ± SE = − 0.010 ± 0.005; *p* = 0.041), malnutrition status (β coefficient ± SE = − 0.129 ± 0.051; *p* = 0.012), and log ferritin level (β coefficient ± SE = − 0.107 ± 0.031; *p* = 0.001) in forward stepwise multiple linear regression analysis (Table 2).

**Table 2:** Determinants of SCL in studied patients (*n* = 647)

	Simple linear regression analysis	*p*	Forward stepwise multiple linear regression analysis	*p*
Variables	Coefficient ± SE		β coefficient ± SE	
Age (years)	−0.004 ± 0.001	0.002		
Body mass index (kg/m^2^)	−0.011 ± 0.005	0.024	− 0.010 ± 0.005	0.041
Hemodialysis vintage (years)	0.005 ± 0.003	0.050		
Diabetes mellitus (Yes = 1)	−0.096 ± 0.036	0.008		
Albumin of < 3.6 g/dL (Yes = 1)	−0.131 ± 0.051	0.011	− 0.129 ± 0.051	0.012
Hemoglobin (mg/dL)	0.041 ± 0.013	0.002		
Creatinine (mg/dL)	0.014 ± 0.007	0.042		
Transferrin saturation (%)	−0.003 ± 0.001	0.020		
Log ferritin (pg/mL)	−0.111 ± 0.033	0.001	− 0.107 ± 0.031	0.001

Abbreviations: *Log* logarithmic transformation, *SE* standard error, *SCL* serum chromium level

### Probability of malnutrition in MHD patients

In univariate logistic regression analysis, we found that the following variables were potential predictors of malnutrition: age of > 65 years; DM; catheter use for blood access; cholesterol, triglyceride, hemoglobin, creatinine, and phosphate levels; nPCR; log SCL; and hsCRP of > 3 mg/L. After adjusting for potential variables, we revealed that age of > 65 years (OR = 1.80; 95% CI: 1.02–3.19; *p* = 0.043) and hsCRP of > 3.0 mg/L (OR = 2.15; 95% CI: 1.22–3.82; *p* = 0.009) were independent and positive predictors of malnutrition, but cholesterol (OR = 0.99; 95% CI: 0.98–1.00; *p* = 0.005), creatinine (OR = 0.73; 95% CI: 0.64–0.84; *p* < 0.001) and log SCL (OR = 0.49; 95% CI: 0.25–0.96; *p* = 0.039) were independent and negative predictors of malnutrition in forward stepwise multivariate logistic regression analysis (Table 3).

**Table 3:** Probability of malnutrition (serum albumin level < 3.6 g/dL) in studied patients (*n* = 647)

	Univariate logistic regression analysis	*p*	Forward stepwise multivariate logistic regression analysis	*p*
Variables	Odds ratio (95% confidence interval)		Odds ratio (95% confidence interval)	
Age > 65 years (Yes vs. No)	3.40 (2.09–5.55)	< 0.001	1.80 (1.02–3.19)	0.043
Diabetes mellitus (Yes vs. No)	1.71 (1.04–2.79)	0.033		
Using catheter as blood access (Yes vs. No)	3.66 (1.35–10.0)	0.011		
Cholesterol (mg/dL) (each increase, 1 mg/dL)	0.98 (0.98–0.99)	< 0.001	0.99 (0.98–1.00)	0.005
Triglyceride (mg/dL) (each increase, 1 mg/dL)	1.00 (0.99–1.00)	0.032		
Hemoglobin (g/dL)	0.55 (0.45–0.69)	< 0.001		
Creatinine (mg/dL) (each increase, 1 mg/dL)	0.65 (0.58–0.74)	< 0.001	0.73 (0.64–0.84)	< 0.001
Phosphate (mg/dL) (each increase, 1 mg/dL)	0.67 (0.55–0.82)	< 0.001		
nPCR (g/kg/day)	0.45 (0.22–0.93)	0.030		
Log SCL (μg/L) (each increase, 10 μg/L)	0.46 (0.25–0.83)	< 0.001	0.49 (0.25–0.96)	0.039
hsCRP of > 3.0 mg/L (Yes vs. No)	2.90 (1.74–4.84)	< 0.001	2.15 (1.22–3.82)	0.009

Abbreviations: *Log* logarithmic transformation, *hsCRP* high sensitivity C-reactive protein, *nPCR* normalized protein catabolic rate, *SCL* serum chromium level

### Probability of inflammation in MHD patients

**Fig. 1:**
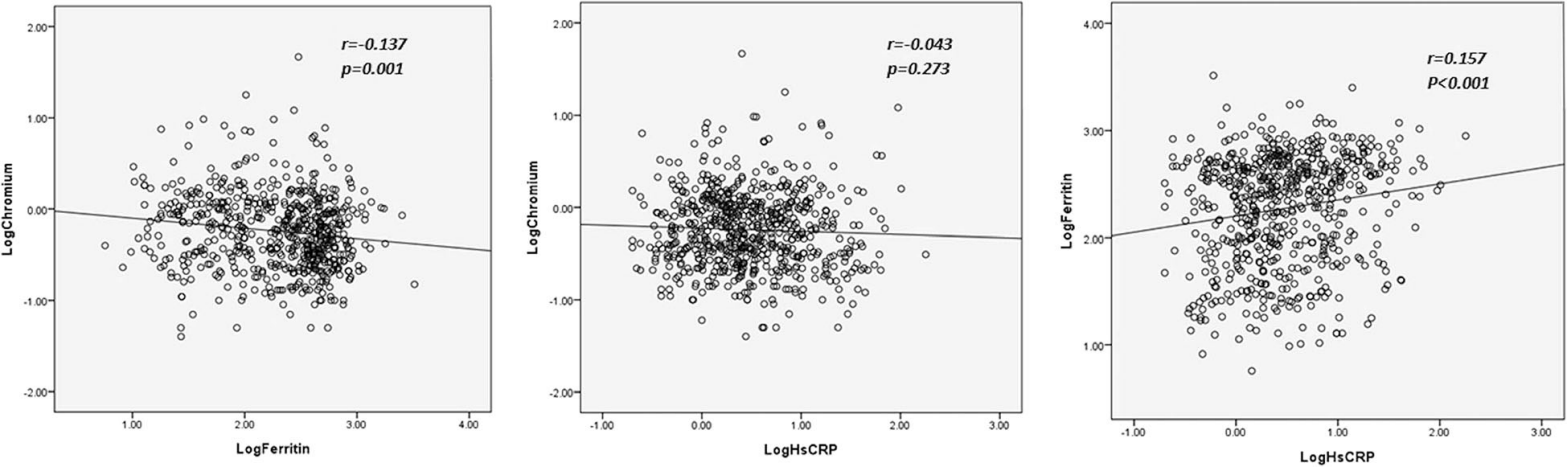
Relationship between logarithmic transformation of hsCRP, ferritin, and chromium. The custom equations were as follows: Log hsCRP = **−** 0.056 [Log chromium] + 0.463, (*p* = 0.273); Log ferritin = − 0.168 [Log chromium] + 2.23, (*p* = 0.001); Log hsCRP = 0.164 [Log ferritin] + 0.096, (*p* < 0.001). Abbreviations: hsCRP, high-sensitivity C-reactive protein; Log, Logarithmic transformation

In univariate logistic regression analysis, we found that the following variables were potential predictors of inflammation: age of > 65 years; body mass index; DM; malnutrition status; catheter use for blood access; Kt/V (Daugirdes); nPCR; and triglyceride, hemoglobin, creatinine, log ferritin, and transferrin saturation levels, but not log SCL (OR = 0.92; 95% CI: 0.64–1.33; *p* = 0.653). After adjusting for potential variables, we revealed that body mass index (OR = 1.15; 95% CI: 1.08–1.22; *p* < 0.001), triglyceride (OR = 1.00; 95% CI: 1.00–1.01; *p* = 0.001), log ferritin (OR = 1.95; 95% CI: 1.28–2.99; *p* = 0.002) and malnutrition (OR = 2.96; 95% CI: 1.61–5.43; *p* < 0.001) were independent and positive predictors of inflammation, but hemoglobin (OR = 0.80; 95% CI: 0.67–0.95; *p* = 0.012) and transferrin saturation (OR = 0.96; 95% CI: 0.94–0.97; *p* = 0.001) were independent and negative predictors of inflammation in forward stepwise multivariate logistic regression analysis (Table 4). The correlation among log hsCRP, ferritin, and chromium is shown in Fig. 1.

**Table 4:** Probability of inflammation (hsCRP > 3.0 mg/L) in studied patients (*n* = 647)

	Univariate logistic regression analysis	*p*	Forward stepwise multivariate logistic regression analysis	*p*
Variables	Odds ratio (95% confidence interval)		Odds ratio (95% confidence interval)	
Age > 65 years (Yes vs. No)	1.68 (1.18–2.39)	0.004		
Body mass index (kg/m^2^) (each increase, 1 kg/m^2^)	1.14 (1.08–1.20)	< 0.001	1.15 (1.08–1.22)	< 0.001
Diabetes mellitus (Yes vs. No)	1.96 (1.39–2.75)	< 0.001		
Using catheter as blood access (Yes vs. No)	2.67 (1.00–7.14)	0.011		
Kt/V (Daugirdes) (each increase, 1)	0.35 (0.21–0.59)	< 0.001		
nPCR (each increase, 1 g/kg/day)	0.57 (0.37–0.87)	0.010		
Triglyceride (mg/dL) (each increase, 1 mg/dL)	1.00 (1.00–1.01)	< 0.001	1.00 (1.00–1.01)	0.001
Hemoglobin (g/dL)	0.81 (0.71–0.93)	0.002	0.80 (0.67–0.95)	0.012
Creatinine (mg/dL) (each increase, 1 mg/dL)	0.93 (0.87–0.99)	0.022		
Transferrin saturation (%) (each increase, 1%)	0.97 (0.95–0.98)	< 0.001	0.96 (0.94–0.97)	0.001
Log Ferritin (each increase, 10)	1.62 (1.18–2.22)	0.003	1.95 (1.28–2.99)	0.002
Albumin of < 3.6 g/dL (Yes vs. No)	2.91 (1.74–4.83)	< 0.001	2.96 (1.61–5.43)	< 0.001

Abbreviations: *Log* logarithmic transformation, *hsCRP* high sensitivity C-reactive protein, *nPCR* normalized protein catabolic rate

## Discussion

The analytical results of this study demonstrated an association between SCLs and malnutrition (serum albumin level < 3.6 g/dL) in MHD patients. Following adjustment for potential variables, SCLs were negatively correlated with malnutrition in these patients. Overall, each 10-fold increase in SCL was associated with a 0.49-fold decrease in the probability of malnutrition development in these subjects. Reviewing the published articles, this study is the first to reveal the negative correlation between SCL and malnutrition in ESRD population.

Chromium, one of the 9 trace elements (iron, zinc, copper, manganese, chromium, cobalt, selenium, fluorine, and iodine) has been reported to be beneficial and essential for humans [1, 2]. The content of chromium in different foods varies widely, and is dependent on chromium introduced in the growing, transport and processing of the foods [16]. Chromium deficiency has been observed in humans under special conditions, such as with long-term total parenteral nutrition supplementation [17] and severe protein-calorie malnutrition [18]. Chromium is excreted primarily in the urine through glomerular filtration [19]. It is not a surprise that chromium levels have been shown to be elevated in the serum of ESRD population [7, 20]. Moreover, although studies indicated that exposure to chromium is a precipitating factor for development of chronic kidney disease in general population [21, 22], no report exists regarding the correlation between SCL and prognosis in MHD patients. A future study is needed to determine whether SCL can serve as a prognostic marker in this population. A similar *study is* currently *underway* in our hospital.

In patients undergoing MHD, disturbances of trace element metabolism might occur because of (1) alterations in gastrointestinal absorption, (2) affected appetite by uremia, and (3) transport during the dialysis procedure [23]. Theoretically, substances with lower concentrations in the dialysate than in the blood tend to be removed by dialysis. On the contrary, substances with higher concentrations in the dialysate than in the blood tend to increase in the blood. However, in most patients, dialysis is an unlikely cause of trace element deficiencies, possibly because the trace elements in the blood have formed a complex with cellular components and proteins [24]. However, chronic toxicity may be a potential hazard if the water supply or dialysis equipment is contaminated. In this study, the chromium levels of all the water and dialysate samples (*n* = 12) were < 0.1 μg/L (far below the AAMI standards [chromium, < 14 μg/L]). After excluding the abovementioned possibilities secondary to dialysis, diet has the largest effect on the SCL of MHD patients.

Trivalent chromium is an essential nutrient that plays a role in the metabolism of certain sugars, proteins, and fats by potentiating the action of insulin [2, 19]. However, occupational or environmental exposure to hexavalent chromium-containing compounds may cause multi-organ toxicity such as renal damage, asthma, and some cancers [3, 25]. It is worth noting that ingested hexavalent chromium is reduced efficiently to the trivalent form in the gastrointestinal tract [26]. Absorption of trivalent chromium can be facilitated by the type of chromium ingested, as well as the effect of vitamins, proteins, drugs, and other nutritional factors, in combination with starch, ascorbic acid, minerals, oxalate, and amino acids [27–29]. Picolinic and nicotinic acids have also been demonstrated to facilitate the absorption of trivalent chromium through the intestinal wall [30]. It is interesting that Anderson et al. indicated that foods high in potassium, saturated fat, and sodium also tend to be high in chromium [31]. Ingestion of phosphorous, vitamin B6, proteins, and carbohydrates also correlated with the ingestion of chromium. In addition, some amino acids such as histidine and glutamic acid increased the absorption of chromium from the intestine [32]. To our knowledge, dietary restriction of potassium, phosphorous, protein, sodium, and fat is recommended for patients with chronic kidney disease with or without dialysis. However, calculations regarding the ingested amount of chromium in restricted diets of patients with dialysis are lacking. In the general population, the recommended dose of trivalent chromium is 50–200 μg per day [33]. However, without additional chromium supplementation, more than 90% of patients’ daily diets were below this recommended level (< 50 μg/day) [34]. Dietary chromium was also correlated with dietary calories (15 μg chromium/1000 kcal in the typical diet consumed in the United States [31]. From the previously mentioned studies and our findings, we can conclude that there is a positive correlation between nutrition and dietary chromium.

Until now, the normal range and clinical role of serum chromium levels are still worthy of discussion, especially for MHD patients. In normal subjects, serum chromium levels range from 0.038 μg/L to 150 μg/L [35]. Volpe et al. found that the mean serum chromium levels were from 0.26 μg/L to 2.62 μg/L after supplementation with 400 μg/day of chromium in the form of chromium picolinate for 12 weeks [36]. In the current study, the SCL ranged from 0.04 μg/L to 46.3 μg/L, which was within the safe range as previously mentioned. The discrepancy of SCL in these MHD patients may be attributed to the following causes: (1) 31.2% patients had residual daily urine of > 100 mL/day, which would decrease the SCL by urinary excretion of chromium in these patients, (2) diet is one of the main sources of chromium exposure, but there are large individual variations due to different amounts of dietary intake and habits, (3) although all studied patients had been prescribed vitamin B complex (contained nicotinamide, 15 mg/tablet, and vitamin B6, 1 mg/tablet) which could increase the absorption of dietary chromium, some of them had poor drug compliance. A further research is needed to determine the exact causes of the discrepancy of SCL in dialysis patients.

Chronic, low-grade inflammation has been considered a hallmark feature in patients with chronic kidney disease [37], and being contributing to the development of protein-energy wasting, as well as accountable for cardiovascular and all-cause mortality [37]. The elevation of inflammatory markers such as C-reactive protein is often observed in chronic dialysis patients [38]. In an animal study [39], chromium supplement could ameliorate inflammation in the respiratory system of type 2 diabetes rats. In human studies, Saiyed et al. [40] revealed that chromium dinicocysteinate supplementation has beneficial effects on reducing vascular inflammation and oxidative stress compared to placebo; Jamilian et al. [41] found that hs-CRP level is decreased after taking chromium for 8 weeks in women with polycystic ovary syndrome. However, we did not found the association between SCL and inflammation in MHD patients, which may be due to the complicated and different mechanisms of inflammation in dialysis patients as compared with the general population [42]. Nevertheless, we found that in the 4 SCL quartile groups, the serum ferritin level decreased as SCL increased (Table 1), and ferritin levels were negatively associated with SCLs (Table 2 and Fig. 1). A study in rats by Ani et al. [43] showed that the serum concentration of ferritin was reduced by 22% following intraperitoneal injections with chromium (1 mg/kg) for 45 days compared with rats injected with saline alone. A study in men by Lukaski et al. [44] indicated that high-dose and long-term chromium picolinate supplement may predispose an individual to iron deficiency. These reports have suggested that chromium may compete with iron for binding to apo-transferrin, and further influence the related biochemical parameters because of the chemical similarities between the two metal ions [43, 45, 46]. On the basis of this interaction, we may observe the negative association between SCL and ferritin in this clinical study. In addition, after adjustment with ferritin and hemoglobin levels, we did not found a significant trend between the doses of erythropoietin and SCL (*p* = 0.588) in the four subgroups. T*here was also no* statistically *significant relationship* between SCL and the doses of erythropoietin (*p* = 0.680) in these studied patients. Hence, low chromium level may not be a reason for erythropoietin resistance in dialysis population. However, a further study is needed to determine the relationship and to explore the mechanism.

This study has some limitations. First, it was a cross-sectional study with patients recruited from a single institution; therefore, the results may not be applicable to ESRD patients from other hospitals. Additional large-scale, multi-center studies are required to confirm our observations. Second, no acceptable indicators have been identified for determining chromium status because chromium concentrations in biological tissues and fluids do not reflect metabolically active chromium pools in the body [47]. Although evaluation of the chromium content in the bones of ESRD patients is another method for assessing chromium levels [48], this invasive procedure may involve some risks. Third, the mean corpuscular volume levels of red blood cells were not available in this study, the influence of chromium on iron metabolism and erythropoiesis could not be clearly assessed. Finally, we did not verify the valence of the serum chromium. However, studies have indicated that patients’ serum chromium can be considered to be trivalent [29, 49].

## Conclusions

This study is the first to demonstrate that SCLs are negatively associated with malnutrition in MHD patients. Additional studies are required to clarify the role of SCLs in inflammation and related comorbidities in this population.

## Data Availability

The datasets used and/or analyzed during the current study are available from the corresponding author on reasonable request.

## Abbreviations

AAMI: American Association for Advancement of Medical Instrumentation
CI: Confidence interval
CVD: Cardiovascular disease
ESRD: End-stage renal disease
HD: Hemodialysis
hsCRP: high-sensitivity C-reactive protein
iPTH: intact parathyroid hormone
MHD: Maintenance hemodialysis
nPCR: normalized protein catabolism rate
OR: Odds ratio
SCL: Serum chromium level
